# LncRNA LBX2-AS1 impacts osteosarcoma sensitivity to JQ-1 by sequestering miR-597-3p away from BRD4

**DOI:** 10.3389/fonc.2023.1139588

**Published:** 2023-03-24

**Authors:** Jiayu Li, Xuhui Yuan, Cong Ma, Junhong Li, Gaoyang Qu, Bo Yu, Feng Cai, Yuanxiang Peng, Lang Liu, Duo Zeng, QuanHui Jiao, Jiongfeng Zhang, Xiaohui Luo, Qi Liao, Xiao-Bin Lv

**Affiliations:** ^1^ Jiangxi Key Laboratory of Cancer Metastasis and Precision Treatment, Central Laboratory, The First Hospital of Nanchang, The Third Affiliated Hospital of Nanchang University, Nanchang, Jiangxi, China; ^2^ Department of Orthopedics, The First Hospital of Nanchang, The Third Affiliated Hospital of Nanchang University, Nanchang, Jiangxi, China; ^3^ Department of Orthopedics, Tongji Hospital, Tongji Medical College, Huazhong University of Science and Technology, Wuhan, Hubei, China

**Keywords:** osteosarcoma, lncRNA LBX2-AS1, miR-597-3p, BRD4, JQ-1

## Abstract

**Objective:**

Recent knowledge concerning the significance of long non-coding RNA (lncRNA)-mediated ceRNA networks provides new insight into their possible roles as specific biomarkers for the treatment of osteosarcoma (OS). Thus, this study aims to clarify the functional relevance and mechanistic actions of lncRNA LBX2-AS1 in OS.

**Methods:**

Differential analysis was performed by integrating the TCGA and GTEx databases. Cox regression analysis was then employed to assess the prognostic value of the model. The expression of lncRNA LBX2-AS1 and miR-597-3p was quantified in OS cell lines by qRT-PCR. The proliferation, migration, invasion, and apoptosis of OS cell lines in response to manipulated lncRNA LBX2-AS1 were evaluated by MTT, colony formation, transwell, Western blot, and flow cytometry assays. Luciferase activity was assayed to validate the reciprocal regulation between lncRNA LBX2-AS1 and miR-597-3p. The protein levels of BRD4 and EMT-related factors were examined by Western blot assay. Finally, tumor growth in response to LBX2-AS1 knockdown was evaluated in xenograft-bearing nude mice.

**Results:**

By integrating the GTEx and TCGA databases, we identified 153 differentially expressed lncRNAs. Among them, 5 lncRNAs, RP11-535M15.1, AC002398.12, RP3-355L5.4, LBX2-AS1, and RP11.47A8.5, were selected to establish a model, which predicted the prognosis of OS. Higher lncRNA LBX2-AS1 expression was noted in OS tissues relative to that in normal tissues. Silencing lncRNA LBX2-AS1 facilitated apoptosis and curtailed proliferative, migratory, and invasive capacities of OS cells. Mechanistically, lncRNA LBX2-AS1 could elevate the expression of BRD4, an oncogene, by competitively binding to miR-597-3p. More importantly, knockdown of lncRNA LBX2-AS1 increased the sensitivity of OS cells to the BRD4 inhibitor JQ-1. Finally, the tumor growth of OS cell xenografts was constrained *in vivo* in the presence of lncRNA LBX2-AS1 knockdown.

**Conclusion:**

In conclusion, lncRNA LBX2-AS1 promotes the growth of OS and represses the sensitivity to JQ-1 by sponging miR-597-3p to elevate the expression of BRD4.

## Introduction

Osteosarcoma (OS), derived from mesenchymal cells, represents a primary malignant bone tumor with an alrmingly high risk of metastatic potential (1). Notably, the age of individuals suffering from OS shows a bimodal distribution (2, 3). The first peak occurs in adolescents experiencing rapid growth and development, and the second peak occurs after the age of 60. The majority of cancers arise in the metaphysis of long tubular diaphyses (1, 4), such as the proximal tibia, distal femur, and distal humerus. OS prognosis is highly connected to the presence of distant metastases (5). Following neoadjuvant treatment and chemotherapy, 78% of adolescents and young adults with locally advanced illnesses survived for five years. Twenty percent of individuals diagnosed with metastasis or recurring metastases have a five-year survival rate (6). The most common metastatic site of OS has been reported to be the lung (7). Notably, because of the limitations of clinical examination procedures, metastases can only be recognized in 15% - 20% of patients (8). Therefore, it is essential to investigate the molecular network controlling metastasis to enhance patient prognosis and early diagnosis.

The central dogma states that genes are transcribed into RNA, and then proteins are encoded from RNA. Proteins are the final products of genetic information, although just 2% of the genome contains protein-coding genes (9, 10). The rest of the genome is composed of non-coding RNAs (ncRNAs), which have not been appreciated for some time. Increasingly, ncRNAs are involved in such critical processes as chromatin remodeling, transcriptional regulation, posttranscriptional modification, as well as signal transduction in many cells (11, 12). Long noncoding RNAs (lncRNAs), as ncRNAs longer than 200 nucleotides, function as translational modulators to modulate mRNA translation. Of interest, lncRNAs serve as “miRNA sponges” (13), which highlights a possible mechanism behind the growth of a wide array of cancers. The competitive endogenous RNA (ceRNA) network dysregulation in OS may result in tumorigenesis (14). For instance, KCNQ1OT1 acts as a miR-34c-5p sponge to enhance aggressiveness of OS (15). LncRNA ODRUL stimulates the development of OS *via* the lncRNA ODRUL/miR-3182/MMP2 axis (16). Therefore, it is critical to note the prominent roles that lncRNAs confer in the prognosis and therapy of OS.

In this work, we hypothesized that OS tissue and normal tissue may express a large number of previously unrecognized lncRNAs. After obtaining all differentially expressed lncRNAs on the basis of differential analysis, we constructed a predictive survival model, which included five lncRNAs. Among them, lncRNA LBX2-AS1 has been documented to exert important effects on carcinogenesis. For example, proliferative potential of gastric cancer has been suggested to be promoted by ectopic expression of the lncRNA LBX2-AS1 (17). However, the mechanistic basis underlying the abnormally elevated lncRNA LBX2-AS1 in OS has not yet been identified.

## Materials and methods

### Bioinformatic prediction

The transcriptome data of 88 samples of OS were retrieved from The Cancer Genome Atlas (TCGA) database (available at https://cancergenome.nih.gov/). The data of 396 cases of normal bone tissues were downloaded from The Genotype-Tissue Expression (GTEx) database (available at www.gtexportal.org). These data were integrated and logarithmically processed (log2 (x + 1)).

### Construction of the predictive risk model

By analyzing the integrated dataset using limma and survival packages, we selected 8 lncRNAs, with p values less than 0.05, through univariate Cox regression analysis for further analyses. After using the LASSO method for analysis, we performed multivariate Cox analysis on the 6 lncRNAs that were selected again. After the above analysis, we obtained a risk scoring model composed of 5 lncRNAs. The formula for the model is Risk score = $\sum_{k=1}^{n}\;$ expression (lncRNA^k^) × coefficient (lncRNA^k^). In this study, the risk formula obtained was Risk score = RP11-535M15.1 × (0.948476561204842) + AC002398.12 × (4.8419) + RP3-355L5.4 × (4.9124) + lncRNA LBX2-AS1 × (0.3376) + RP11.47 A8.5 × (−0.1294). The patients were grouped on the basis of the calculated median risk score, followed by plotting of survival curves.

### Cell lines

The OS cell lines (ZOS, U2R, U2OS, MG63, and 143B) in this study were conserved in our laboratory. OS cells were cultured in DMEM (Solarbio) with 10% fetal bovine serum (FBS, Gibco, Grand Island, NY, USA) with 5% CO_2_ and high saturated humidity at 37°C.

### Plasmid construction and cell transfection

The lncRNA LBX2-AS1 fragment and the 3’UTR of the BRD4 fragment containing the predicted miR-597-3p binding sites were cloned into the pmirGLO dual-luciferase reporter vector. The mutation was carried out using a Q5^®^ Site-Directed Mutagenesis Kit (New England Biolab) as per the manufacturer’s protocols.

The small interfering RNAs (siRNAs) targeting lncRNA LBX2-AS1 were designed and synthesized by GenePharma (Shanghai, China), and miRNA mimics were obtained from the same manufacturer. The siRNAs and mimics were subjected to transfection by Lipofectamine RNAiMAX (Invitrogen, USA) based on polyethyleneimine (PEI, Polyscience, Illinois, USA). The siRNA sequences are listed below:

LncRNA LBX2-AS1#1 F: 5’-GCUGCUCGGUCCCUGCAAATT-3’,

R: 5’-UUUGCAGGGACCGAGCAGCTT-3’.

LncRNA LBX2-AS1#2 F: 5’-GCUACCUUCAGGAAUUCUUTT-3’,

R: 5’-AAGAAUUCCUGAAGGUAGCTT-3’.

The shRNA sequence against lncRNA LBX2-AS1 cloned into the pLKO.1 plasmid was co-transfected with helper vectors into 293T cells for 48 h, and the recombinant virus delivering lncRNA LBX2-AS1 shRNA was collected. The recombinant virus was then transduced into OS cells, with the positive cells identified by puromycin (2 μg/ml) culture for 2 weeks.

### MTT-based method

Cell viability was observed using the MTT assay. Cells seeded in 96-well plates (1 × 10^3^ cells/well) were added to MTT solution (Solarbio, China) at working concentrations in a dark environment for 4 h. Then, 100 µl of DMSO solution was supplemented to dissolve formazan crystals. The optical density (OD) value at 490 nm was observed using a microplate reader (Bio-Rad, Hercules, CA, USA).

### Transwell assay

Transwell chambers coated or uncoated with Matrigel (Thermo Fisher Scientific, USA) were employed to assess *in vitro* cell migratory and invasive capacities. The upper chamber was plated with 1 × 10^5^ cells resuspended in FBS-free medium (200 μl). It was then placed in a 24-well plate with complete medium (500 μl) in the bottom chamber. After incubation for approximately 20 h, cells were immobilized with methanol and stained with 0.1% crystal violet. After removing the un-penetrated cells using a cotton swab, the penetrated cells were quantified by ImageJ software and images were acquired under a microscope (Olympus, Japan).

### Colony formation assay

Colony formation of cells was assayed in 6-well plates, with 500 cells in each well. The medium supplemented with 10% FBS was renewed every 2 days. After incubation of 12 days, the cells were fixed with methanol, stained with 0.1% crystal violet, and photographed to count colonies.

### Apoptosis assay

The apoptosis was assayed using an Apoptosis Detection Kit (KeyGEN BioTECH, KGA108-1) as instructions described. Cells in the silencing group and control group were collected after 48 h of culture. Then, the cells were suspended in binding buffer, followed by staining with 1% propidium iodide (PI) and 1% annexin V-FITC for 30 min without light exposure. The quantification of apoptotic cells was conducted by flow cytometry (Becton-Dickinson, USA).

### RNA extraction and qRT-PCR verification

After RNA extraction by TRIzol reagents, cDNA was reverse transcribed with HiScript II Q RT SuperMix (R223-01, Vazyme). Real-time quantitative PCR was performed on a CFX96 Real-Time PCR Detection System using ChamQ Universal SYBR qPCR Master Mix (Vazyme, Q711-02). As normalized to GAPDH and U6, the data were processed using the 2^−ΔΔCt^ method. The primer sequences are listed as follows:

LncRNA LBX2-AS1 F: 5’-AGTTTGTCCCAGGTTTGGCA-3’,

R: 5’-CATGCCAGGGTCCTTGTTCT-3’.

miR-597-3p F: 5’-ACACTCCAGCTGGGtGGttCtCttGtGGCtC-3’,

R: 5’-TGGTGTCGTGGAGTCG-3’.

GAPDH F: 5’-GGAGCGAGATCCCTCCAAAAT-3’,

R: 5’-GGCTGTTGTCATACTTCTCATGG-3’.

U6 F: 5’-GACTATCATATGCTTACCGT-3’,

R: 5’-GGGCAGGAAGAGGGCCTAT-3’.

### Luciferase reporter assay

The binding site of lncRNA LBX2-AS1 to miR-597-3p was predicted using tools of LncBase (available at https://dianalab.e-ce.uth.gr/html) and LncRNASNP2 (available at http://bioinfo.life.hust.edu.cn/lncRNASNP). The prediction of putative genes targeted by miR-597-3p was carried out using the TargetScan 8.0 database (www.targetscan.org). The miR-597-3p mimics or miRNA-NC were co-transfected for 48 h with LBX2-AS1-wild-type (WT) or LBX2-AS1-mutant (Mut) plasmids into 293T cells. Luciferase activities were determined based on the Dual Luciferase Reporter Assay System (Promega, USA). To validate the binding of miR-597-3p to BRD4, WT or Mut BRD4 3’UTR fragments cloned in the pmirGLO plasmids were co-transfected for 48 h with miR-597-3p mimics or miRNA-NC into 293T cells. Luciferase reporter analysis was carried out using the Dual-Lumi™ Luciferase Reporter Gene Assay Kit (Beyotime, Shanghai, China).

### Western blot analysis

Cells were lysed in cold RIPA buffer (50 mM Tris–HCl, 150 mM NaCl, 5 mM EDTA, 0.5% Nonidet P-40) containing protease inhibitor cocktail (Calbiochem, San Diego, CA, USA) on ice for 30 min. Lysates were centrifuged at 14,000 rpm at 4°C for 30 min. Protein concentration of the harvested supernatants was quantified with a BCA kit (Beyotime). The proteins were subjected to SDS-PAGE and transferred onto polyvinylidene difluoride (PVDF) membranes (Burlington, MA, USA). After being blocked with 5% skim milk powder, the membranes were probed with specific primary antibodies at 4°C overnight. Following three washes in TBS solution (Solarbio, China), the membrane was incubated with horseradish peroxidase-conjugated secondary antibody (1:10000, Promega, USA) for 1 h at ambient temperature. Immunocomplexes were visualized using an ECL kit (Tiangen, China) and quantified using the ImageJ software. The antibodies included anti-GAPDH (Proteintech, China; internal control), anti-vimentin (Abcam, Cambrige, UK), anti-BRD4 (Cell Signaling Technology, USA), anti-N-cadherin (Abcam), and anti-E-cadherin (Abcam).

### Xenograft models

Twelve 4-6-week-old BALB/C nude mice (Shanghai Institutes for Biological Sciences, Shanghai, China) were randomly grouped (6 mice per group). The nude mice were injected subcutaneously with MG63 cells stably transfected with sh-LBX2-AS1-1 or sh-NC. The tumor volumes were measured at designated time points (5, 10, 15, 20, and 25 days). The mice were sacrificed on Day 25, with tumors removed and weighed.

### Statistical analyses

All results are shown as the mean ± standard deviation (SD), and the cell function experiments were performed three times. Statistical significance was assessed by one-way ANOVA (multi-group comparisons) and Student’s t test (two-group comparisons). The difference was regarded significant at *p*< 0.05.

## Results

### LncRNA-associated risk models in the prognosis of OS

**Figure 1:**
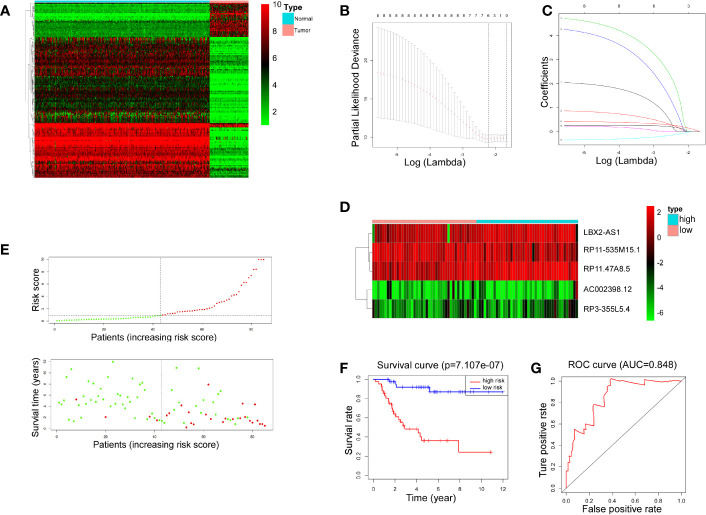
LncRNA-associated risk models’ clinical predictive value. **(A)** Heatmap representing 153 differentiallly expressed lncRNAs between 88 OS tissues and 396 normal tissues. Red indicates increased expression and green indicates decreased expression. **(B, C)** Six lncRNAs were screened by univariate regression and LASSO analysis. **(D)** Heatmap showing expression levels of 5 lncRNAs selected by multiple regression analysis. **(E)** Scatter plots and risk curves sorted by risk score from low to high. **(F)** Overall survival curves of high-risk and low-risk groups (p< 0.001). **(G)** The receiver operating characteristic curve has an AUC of 0.848.

The transcriptional data from OS tissue and normal tissue were downloaded from the TCGA and GTEx databases, respectively. Differential analysis was performed using the R programming language. A total of 153 differentially expressed lncRNAs were identified for the next step of analysis (**Figure 1A**). According to univariate regression analysis and LASSO analysis, we screened six lncRNAs (RP11.211G3.2, RP11.535M15.1, AC002398.12, RP3.355L5.4, LBX2.AS1, and RP11.47A8.5), which were associated with the prognosis of OS (**Figures 1B, C**). After multiple regression analysis, five of these lncRNAs (RP11-535M15.1, AC002398.12, RP3-355L5.4, LBX2-AS1, and RP11.47A8.5) were selected as prognostic markers (**Figure 1D**).

Furthermore, 88 patients were partitioned, according to the risk score, into high-risk and low-risk groups. The scatter plots and risk curves suggested that the mortality rate of OS patients increased with increasing risk score (**Figure 1E**). According to KM (Kaplan-Meier) survival analysis, an appreciable difference (*p*< 0.001) was witnessed in overall survival (OS) between the two groups, indicating that the prognostic model composed of 5 lncRNAs had a promising clinical prediction effect (**Figure 1F**). The area under the curve (AUC) was 0.848 on the basis of ROC curve analysis (**Figure 1G**), further confirming the predictive value of the risk model.

### Knocking down lncRNA LBX2-AS1 reduces malignant phenotypes and increases apoptosis of OS cells

**Figure 2:**
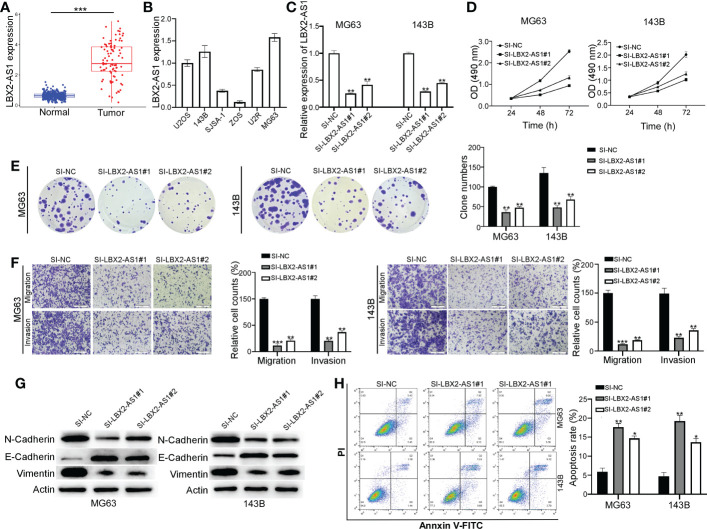
Knocking down the lncRNA LBX2-AS1 in OS cells reduces proliferation and metastasis and increases apoptosis. **(A)** The expression level of lncRNA LBX2-AS1 was extracted from the dataset of the TCGA database combined with the GTEx database. **(B)** Expression levels of lncRNA LBX2-AS1 in six OS cell lines. **(C)** Detection of the efficiency of silencing lncRNA LBX2-AS1 by qRT-PCR. **(D, E)** Detection of Cell Viability Using MTT and Clonogenic Assays. **(F)** Transwell migration assay measures the metastatic ability of cells. Scale bar = 50 μm. **(G)** The protein expressions of N-cadherin, E-cadherin, and vimentin were analyzed by Western blot. **(H)** Detection of apoptosis in transfected cells by flow cytometry. *p< 0.05; **p< 0.01;*** p< 0.001.

The tumor-promoting role of lncRNA LBX2-AS1 has been suggested in multiple malignancies (18–20), though the mechanistic actions remain poorly understood. As expected, its expression was found to be abnormally increased in OS (**Figure 2A**), which was further verified in different OS cell lines (U2OS, 143B, SJSA-1, ZOS, U2R, and MG63) by qRT-PCR (**Figure 2B**). MG63 and 143B cells, which had relatively higher levels of lncRNA LBX2-AS1, were transfected with siRNAs (SI-NC, SI-LBX2-AS1#1, and SI-LBX2-AS1#2), with the silencing efficiency experimentally confirmed (**Figure 2C**).

Based on the results of the MTT and colony formation assays, silencing lncRNA LBX2-AS1 diminished the proliferative ability of OS cells (**Figures 2D, E**). Also, the migratory and invasive abilities of MG63 cells and 143B cells were blunted in response to lncRNA LBX2-AS1 knockdown, as reflected by Transwell assays (**Figure 2F**). Subsequently, the role of lncRNA LBX2-AS1 was further examined by analyzing epithelial-mesenchymal transition (EMT) of OS cells. It was observed that silencing lncRNA LBX2-AS1 diminished the levels of N-cadherin and vimentin (mesenchymal markers), and elevated the levels of E-cadherin (epithelial marker) (**Figure 2G**). In addition, the OS cell apoptosis was enhanced by lncRNA LBX2-AS1 knockdown, as reflected by flow cytometric assay (**Figure 2H**). Collectively, the data suggest that silencing lncRNA LBX2-AS1 curtails cell proliferation, metastasis, and resistance to apoptosis of OS cells.

### LncRNA LBX2-AS1 is a molecular sponge of miR-597-3p

**Figure 3:**
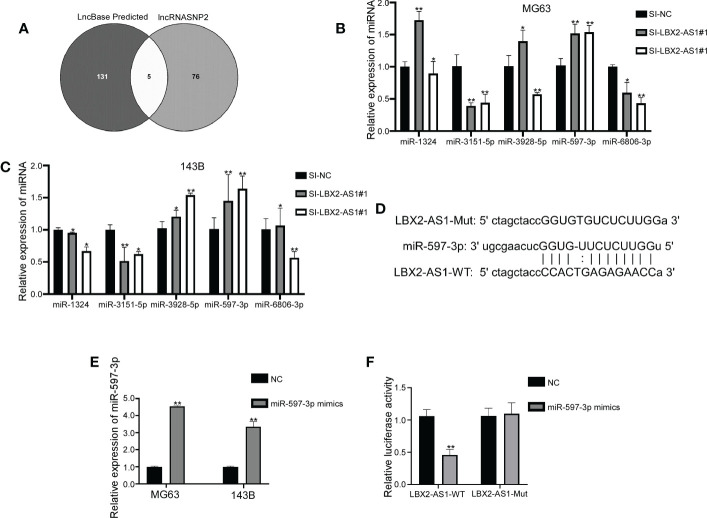
LncRNA LBX2-AS1 is a molecular sponge of miR-597-3p. **(A)** Venn diagram showing miRNAs shared by two prediction sites. **(B, C)** qRT-PCR for the detection of miRNA changes in transfected cells. **(D)** Predicted binding sites for miR-597-3p and lncRNA LBX2-AS1. **(E)** Expression levels of transfected miR-597-3p mimics measured by qRT-PCR. **(F)** Dual-luciferase reporter assay confirming the binding of miR-597-3p and lncRNA LBX2-AS1. *p< 0.05; **p< 0.01.

The downstream mechanisms of lncRNA LBX2-AS1 in OS were further delineated. Putative miRNA targets were singled out using the Lncbase Predicte and lncRNASNP2 databases, with 5 miRNAs identified in both databases (**Figure 3A**). Further qRT-PCR analysis implied that silencing lncRNA LBX2-AS1 elevated the levels of miR-597-3p, without altering levels of other miRNAs in both OS cell lines (**Figures 3B, C**), indicating that miR-597-3p might be a downstream target of lncRNA LBX2-AS1.

The functional interplay between lncRNA LBX2-AS1 and miR-597-3p was further confirmed by a luciferase reporter assay. It was demonstrated that mimics of miR-597-3p diminished the luciferase activity of the LBX2-AS1-WT group. However, mutation of the binding sites in lncRNA LBX2-AS1 abrogated this attenuation of luciferase activity by miR-597-3p mimics (**Figures 3D–F**). Taken conjointly, these data indicate that lncRNA LBX2-AS1 may act as a molecular sponge of miR-597-3p in OS cells.

### LncRNA LBX2-AS1 regulates OS cell functions by sponging miR-597-3p

**Figure 4:**
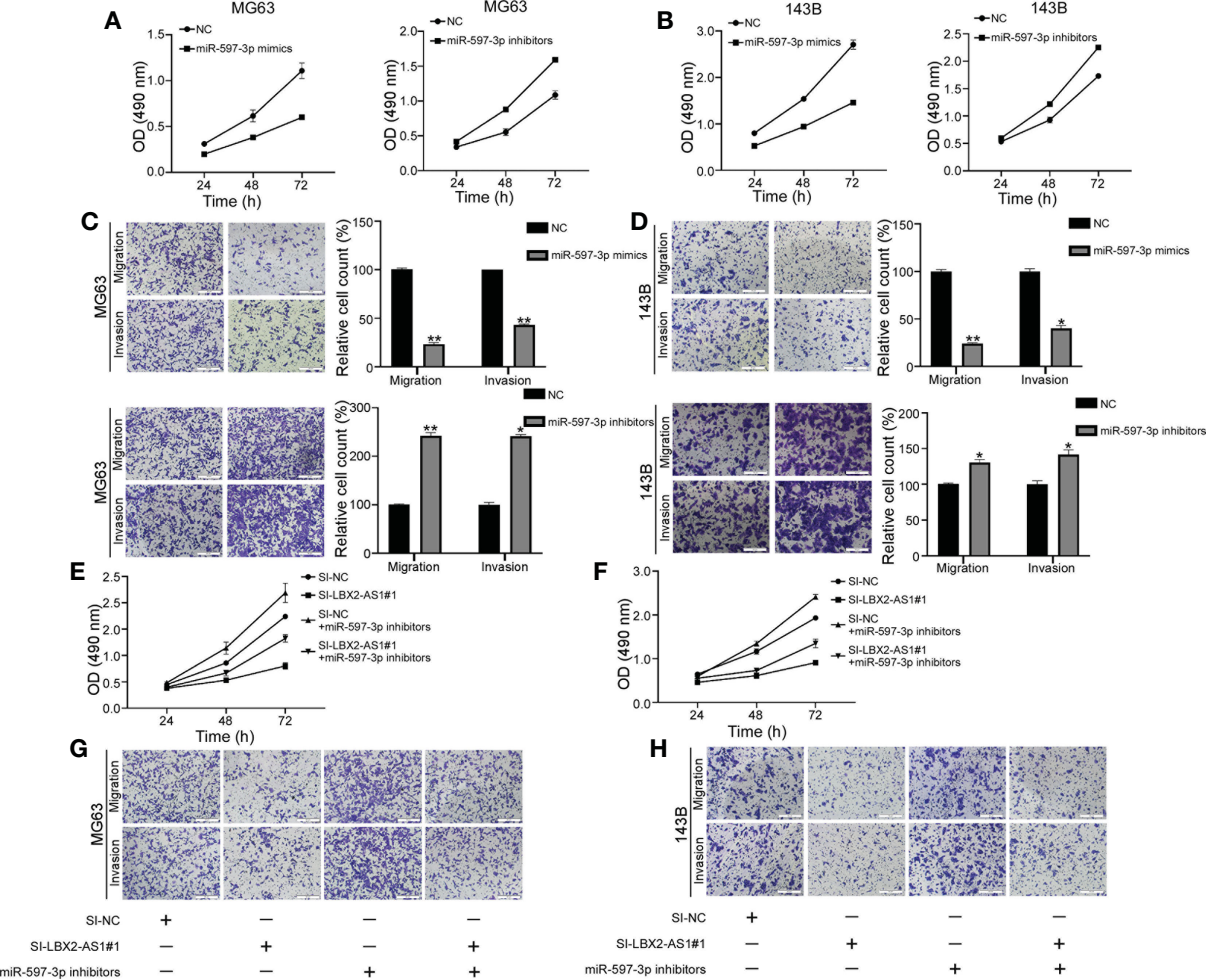
LncRNA LBX2-AS1 regulates OS cell function by sponging miR-597-3p. **(A, B)** An MTT assay was used to examine the proliferative effects of miR597-3p mimics and miR-597-3p inhibitors. **(C, D)** Transwell assays of the metastatic effects of miR-597-3p mimics and miR-597-3p inhibitors. Scale bar = 50 mm. **(E, F)** MTT detection of MG63 and 143B cell proliferation. **(G, H)** Transwell assays were used to detect the metastatic ability of MG63 and 143B cells. Scale bar = 50 mm. *p< 0.05; **p< 0.01.

Evidence exists reporting that miR-597-3p might function as a tumor suppressor (21), which intrigued us to examine the role of miR-597-3p in OS. The MTT assay indicated that the delivery of miR-597-3p mimic diminished OS cell viability, while its inhibitor had the opposite effect (**Figures 4A, B**). Consistently, the migratory and invasive capacities of OS cells were curbed in response to miR-597-3p mimic, which were facilitated by its inhibitor as exhibited by Transwell assays (**Figures 4C, D**). Thus, the tumor-inhibiting role of miR-597-3p in OS was validated, which may be ascribed to the repression induced by lncRNA LBX2-AS1. Indeed, the data of MTT and transwell assays highlighted that miR-597-3p inhibitor counterweighed the suppressive effect of lncRNA LBX2-AS1 knockdown on the malignant phenotypes of OS cells (**Figures 4E–H**). Accordingly, it could be concluded that miR-597-3p is a tumor suppressor and it can be sponged by lncRNA LBX2-AS1.

### BRD4 is a downstream target of miR-597-3p

**Figure 5:**
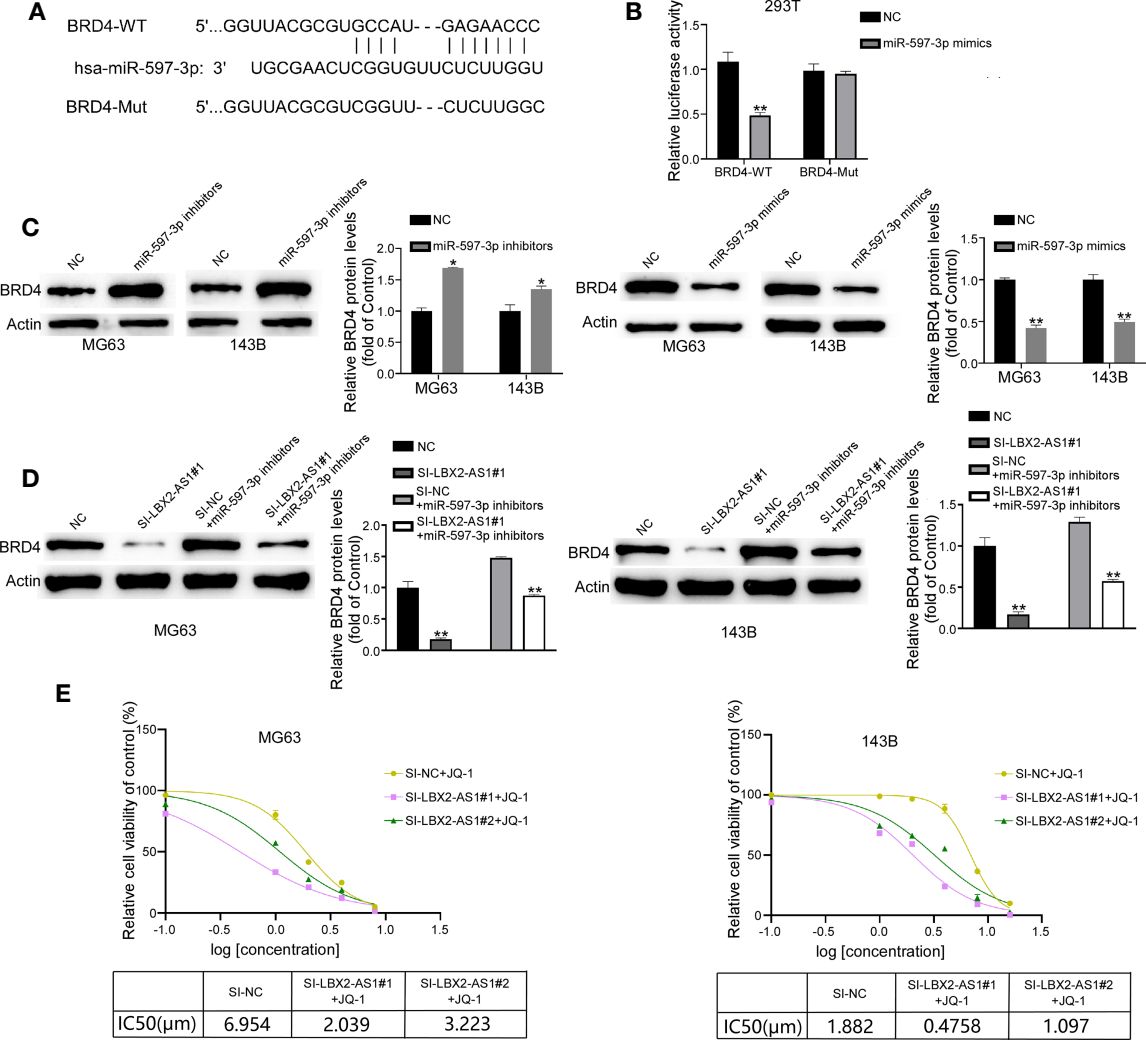
BRD4 is the target of miR-597-3p, and silencing lncRNA LBX2-AS1 increases the sensitivity of OS cells to JQ-1. **(A)** Predicted binding site of miR-597-3p and the BRD4 3’UTR. **(B)** Validation of predicted binding sites by dual-luciferase reporter assay. **(C, D)** The expression level of BRD4 was measured by Western blot. **(E)** MTT assay was used to detect cell viability, and GraphPad was used to calculate the IC50. *p< 0.05; **p< 0.01.

We used the TargetScan 8.0 database to screen the targets of miR-597-3p, which revealed BRD4 as a putative target of miR-597-3p (**Figure 5A**). We constructed luciferase reporter vectors with BRD4-WT-3′UTR or BRD4-MUT-3′UTR containing putative or mutated binding sites. It was demonstrated that miR-597-3p mimics decreased the luciferase activity of the BRD4-WT-3’UTR group, but not the BRD4-MUT-3’UTR group (**Figure 5B**). In addition, the mimic of miR-597-3p diminished the protein level of BRD4 in OS cells, whereas its inhibitor led to opposite results (**Figure 5C**). Indeed, Western blot suggested that miR-597-3p inhibitor reversed the lncRNA LBX2-AS1 knockdown-dependent suppressive effect on the protein level of BRD4 in OS cells (**Figure 5D**). Of interest, BRD4 level has been reported to be associated with the sensitivity of cells to its inhibitor. We hence examined whether modulation of the lncRNA LBX2-AS1 level regulated the sensitivity of OS cells to JQ1, a specific BRD4 inhibitor. Indeed, silencing lncRNA LBX2-AS1 in OS cells obviously decreased the IC50 of JQ1 in OS cells (**Figure 5E**). Collectively, the lncRNA LBX2-AS1/miR-597-3p interplay regulates the protein expression of BRD4, and silencing lncRNA LBX2-AS1 potentiates the sensitivity of OS cells to JQ-1.

### Knockdown of lncRNA LBX2-AS1 inhibits xenograft growth of OS cells *in vivo*

**Figure 6:**
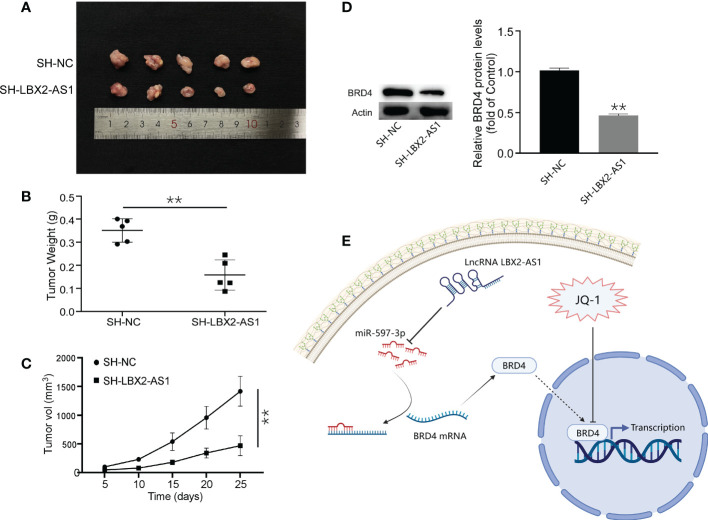
Knockdown of lncRNA LBX2-AS1 inhibited the growth of OS cells *in vivo*. **(A)** Image of a mouse subcutaneous tumor. **(B)** Measuring the weight of the tumor body after 25 days. **(C)** Tumor volume was measured every 5 days. **(D)** The expression of BRD4 was detected by Western blot. **(E)** Experimental Model Diagram Created with Biorender.com. **p< 0.01.

The role of lncRNA LBX2-AS1 was subsequently clarified *in vivo*. A MG63 cell line stably transfected with sh-LBX2-AS1 or sh-NC was developed and injected into nude mice. After 25 days, the tumors in nude mice in response to sh-LBX2-AS1 were smaller than those in the presence of sh-NC (**Figures 6A–C**). Moreover, Western blot analysis for tumor samples revealed a notable downregulation of BRD4 expression in the sh-LBX2-AS1-treated group compared to the control group (**Figure 6D**). In summary, silencing sh-LBX2-AS1 diminished tumor growth in OS cell xenografts.

## Discussion

In recent years, it has become clear that lncRNAs play an increasingly critical role in cancer diagnosis and treatment due to their modulation on the growth and differentiation of tumor cells as well as drug resistance in malignant tumors. For example, lncRNA EIF3J-DT increases ATG14 expression by sequestering miR-188-3p and activates autophagy, leading to drug resistance in gastric cancer cells (22). It suggests the involvement of ceRNA mechanism in the oncogenic role of lncRNAs. Overexpression of lncRNA HOTAIR leads to cisplatin resistance in human lung adenocarcinoma cells (23). It has been reported that lncRNA LBX2-AS1 is a tumor-promoting factor that can promote cell proliferation and metastasis and reduce apoptosis. Examples include glioma (24), ovarian cancer (25), thyroid cancer (26), and gastric cancer (27). LncRNAs have been identified as potential therapeutic targets for treating drug-resistant tumors. However, the mechanism by which lncRNA LBX2-AS1 contributes to OS development has not yet been reported. The present study offers novel mechanistic insights based bioinformatics prediction and experimental validations. Of note, the lncRNA LBX2-AS1/miR-597-3p/BRD4 ceRNA network was involved in the malignant biological characteristics of OS.

Functional experiments revealed the prominent carcinogenic potential of BRD4 in the lncRNA LBX2-AS1/miR-597-3p/BRD4 ceRNA network. BRD4 is a member of the Bromo and extra C-terminal domain (BET) protein family (28). BET proteins affect epigenetic information changes in histones in the development of many diseases by binding to acetylated chromatin *via* bromodomains and, thereby modulating such biological processes as proliferation, differentiation, and apoptosis. For example, inhibition of BRD4 suppresses pyroptosis and alleviates acute gouty arthritis (29). Inhibition of BRD4 reduces RAD51AP1 transcription and sensitizes cervical cancer to radiation (30). JQ-1 is a BET bromodomain inhibitor (31). It can bind to the acetylated lysine recognition pocket of the bromodomain, thereby blocking bromodomain-mediated regulation of acetylation. The clinical development and use of BET bromodomain inhibitors have their challenges, such as dose-limiting toxicity and resistance (32, 33). Utilizing modest dosages of JQ-1 can decrease treatment-related problems and increase patient survival time.

As for the upstream effectors, we identified a predictive model consisting of 5 lncRNAs by multiple bioinformatics analyses and confirmed its predictive significance for the survival outcome of OS patients by ROC curves. Interestingly, *in vitro* experiments of our work highlighted that silencing of the lncRNA LBX2-AS1 augmented cell apoptotic capacity and retarded proliferative, migratory, and invasive capacities. Our findings concur with such findings reported earlier that lncRNA LBX2-AS1 acts as a miRNA sponge to affect the invasion and migration of tumor cells (34, 35). Mechanistic exploration pointed out that lncRNA LBX2-AS1 sequestered miR-597-3p to relieve its repression on BRD4 in OS cells. Additionally, ectopic expression of miR-597-3p inhibited malignant phenotypes of OS cells by diminishing BRD4 levels. More importantly, silencing lncRNA LBX2-AS1 increased the sensitivity of OS cells to JQ-1 and diminished tumor growth *in vivo*. Our experiments suggested that lncRNA LBX2-AS1 facilitated the growth of OS and repressed sensitivity to JQ-1 by sequestering miR-597-3p away from BRD4 (**Figure 6E**).

However, this study has some limitations. First, although bioinformatics analyses provided promising information, the findings need to be further validated and confirmed in larger patient cohorts. Secondly, although the involvement of lncRNA LBX2-AS1/miR-597-3p/BRD4 axis in the sensitivity of JQ-1 was preliminarily predicted and verified *in vitro*, more in-depth experiments are needed *in vivo* to validate this hypothesis in future investigations.

## Conclusion

Data obtained in this study provided new mechanistic insights for an understanding of the role of lncRNA LBX2-AS1 in OS. LncRNA LBX2-AS1 served as a sponge of miR-597-3p, relieving its repression on BRD4 expression. Abnormal expression of the BRD4 protein affects the expression of downstream genes, which then impacts the normal function of cells. Silencing of lncRNA LBX2-AS1 increased the sensitivity of OS cells to the BRD4 inhibitor JQ-1. Therefore, the identification of the lncRNA LBX2-AS1 AS1/miR-597-3p/BRD4 axis may provide a potential target for the treatment of OS patients.

## Data availability statement

The original contributions presented in the study are included in the article/supplementary material. Further inquiries can be directed to the corresponding author.

## Ethics statement

This study was approved by the institutional ethical review boards of the Third Affiliated Hospital of Nanchang University.

## Author contributions

X-BL and QL designed the current study and amended the paper. JYL, XY, and CM were the major writer of the paper, JHL, GQ, and BY created all tables and figures and provided suggestions for important intellectual content. FC, YP, LL, DZ, and QJ performed western blotting and drug treatment experiments. JZ and XL performed the *in vivo* assay. All authors discussed the results. All authors contributed to the article and approved the submitted version.
